# Coproduction of xylo-oligosaccharides and glucose from sugarcane bagasse in subcritical CO_2_-assisted seawater system

**DOI:** 10.1186/s40643-022-00525-3

**Published:** 2022-03-28

**Authors:** Leping Zhang, Xiankun Zhang, Fuhou Lei, Jianxin Jiang, Li Ji

**Affiliations:** 1 Department of Chemistry and Chemical Engineering, MOE Engineering Research Center of Forestry Biomass Materials and Bioenergy, Beijing Forestry University, Beijing, 100083 China; 2 Key Laboratory of Chemistry and Engineering of Forest Products, State Ethnic Affairs Commission, Guangxi Key Laboratory of Chemistry and Engineering of Forest Products, School of Chemistry and Chemical Engineering, Guangxi University for Nationalities, Nanning, 530006 China

**Keywords:** Sugarcane bagasse, Xylo-oligosaccharides, Subcritical CO_2_-assisted seawater pretreatment, Glucose, Enzymatic hydrolysis

## Abstract

Abundant seawater resources can replace the shortage of freshwater resources. The co-production of xylo-oligosaccharides and glucose from sugarcane bagasse by subcritical CO_2_-assisted seawater pretreatment was studied in this paper. We investigated the effects of pretreatment conditions of temperature, CO_2_ pressure and reaction time on the yield of xylo-oligosaccharides in subcritical CO_2_-assisted seawater systems. The maximum xylo-oligosaccharide yield of 68.23% was obtained at 165 °C/2 MPa/5 min. After further enzymatic hydrolysis of the solid residue, the highest glucose yield of 94.45% was obtained. In this system, there is a synergistic effect of mixed ions in seawater and CO_2_ to depolymerize xylan into xylo-oligosaccharides with a lower degree of polymerization. At the same time, the addition of CO_2_ increased the pore size and porosity of sugarcane bagasse, improved the efficiency of enzymatic hydrolysis and increased the yield of glucose. Therefore, this study provides a more environmentally friendly and sustainable process for the co-production of xylo-oligosaccharides and glucose from sugarcane bagasse, and improves the utilization of seawater resources.

**Graphical Abstract:**

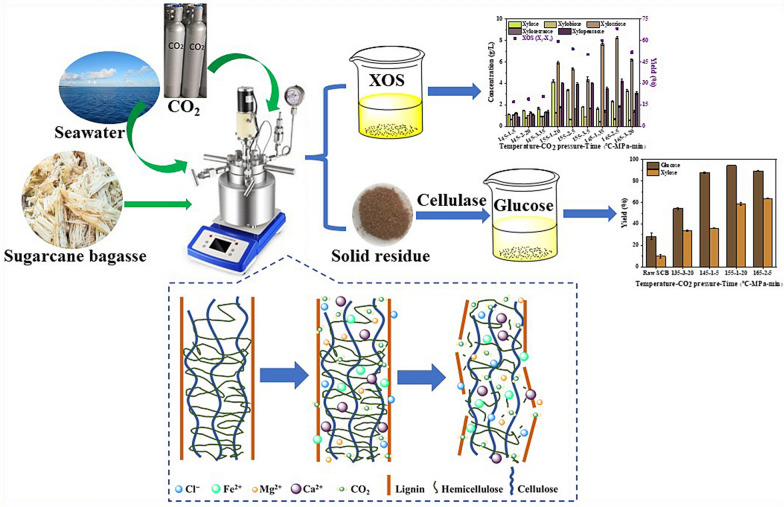

**Supplementary Information:**

The online version contains supplementary material available at 10.1186/s40643-022-00525-3.

## Introduction

The rapid depletion of fossil fuels and severe environmental pollution prompt people to seek sustainable resources to replace non-renewable fossil resources (Gong et al. 2021; Ni et al. 2021). Lignocellulosic biomass is an ideal resource for biofuel and high value-added chemicals production due to its wide distribution, abundant reserves, low cost and renewability (Wu et al. 2021b). As one of the lignocellulosic biomass materials, sugarcane bagasse (SCB) is abundant and consists of three main components: cellulose, hemicellulose and lignin (Ajala et al. 2021). The cell wall structure of SCB is formed by the cross-linking of a lignin–hemicellulose–cellulose (Ire et al. 2016), which makes it challenging to produce biofuels or chemicals directly. Therefore, hunting for a proper pretreatment method is necessary to improve the utilization of hemicellulose, enhance the enzyme digestibility of cellulose and maintain the structural integrity of lignin, achieving the utilization of the whole composition of lignocellulose finally (Liu et al. 2019; Zhang et al. 2020a).

Xylo-oligosaccharide (XOS) is a soluble carbohydrate produced by partial hydrolysis of hemicellulose at the high temperature. It consists of xylose chains of lengths 2–10, with or without arabinose or glucuronic acid side groups (Jaichakan et al. 2021). The biological activity of XOS will depend on their degree of polymerization. According to its different biological activities, XOS is primarily used in the functional food, pharmaceutical and chemical industries (de Freitas et al. 2019).

At present, a large number of pretreatment methods for XOS production have been developed, such as acid or alkaline hydrolysis (Liao et al. 2021a; Yang et al. 2021; Zhang et al. 2021a), autohydrolysis (Zhang et al. 2020b), biological pretreatment (Valladares-Diestra et al. 2021), inorganic salt pretreatment (You et al. 2020; Zhang et al. 2019) and the combination of some of them (Liao et al. 2021b; Nuntawat et al. 2018). These methods have their advantages and disadvantages, but they all have one thing in common: they all use many freshwater resources as the main solvent. However, if XOS is produced on a large scale, it may cause excessive use of freshwater resources. Seawater resources are abundant, accounting for about 97% of the world's water resources. Our previous research has shown that seawater containing a variety of inorganic salt ions can catalyze the production of XOS from SCB (Zhang et al. 2020b). The concentrations of iron, magnesium, calcium and NaCl in the seawater are 1.25 ± 0.01 mg/mL, 414.40 ± 0.20 mg/mL, 199.05 ± 0.05 mg/mL and 27.13 ± 0.00 mg/mL, respectively (Zhang et al. 2020b). The metal ions in these inorganic salts provide an indirect weak acidic (Lewis acid) environment. In this subacidic environment, chloride ions can cut off the hydrogen bond between glycan molecules, to promote the degradation of highly polymerized glycans into smaller polysaccharides or even monosaccharides (Jiang et al. 2018; Zhang et al. 2020b).

During these available pretreatment processes, a large amount of hemicellulose was degraded into XOS and retained in the liquid components, while most cellulose and lignin were still kept in the solid parts (Zhang et al. 2021b, 2020a, 2020b; Zhu et al. 2021). Our previous study demonstrated that the seawater hydrothermal pretreatment of SCB is a highly efficient and environmentally friendly method for XOS production (Zhang et al. 2020b). It combined the advantages of automatic hydrolysis and ion pretreatment, which was green, efficient, lower cost, non-toxic and without additional chemical reagents. Seawater hydrothermal pretreatment can not only obtain a high yield of XOS, but also a high yield of glucose in the enzymatic hydrolysis stage of solid residue. However, in addition to receiving high-yield XOS, the existing pretreatment conditions are still severe (higher temperature and severity parameter). CO_2_ pretreatment is a non-toxic, environmentally friendly, and efficient method for the pretreatment of lignocellulosic biomass (Ge et al. 2020). In the pretreatment process, CO_2_ can form a weak acid (H_2_CO_3_) catalyst with water to degrade hemicellulose and further destroy the lignin–carbohydrate complex (LCC) compound structure. After the reaction, rapid release and separation of CO_2_ can be achieved through decompression (Gurgel et al. 2014; Liu et al. 2019; Park and Lee 2020). The separation methods of XOS include solvent extraction, membrane separation and chromatography, in which chromatography is the most efficient one to obtain high-purity XOS (Xiao et al. 2018). Moreover, the metal ions (iron, magnesium and calcium) contained in seawater are beneficial to human body (Zhang et al. 2018).

Therefore, an effective lignocellulosic pretreatment was proposed using subcritical CO_2_-assisted seawater pretreatment (SCSP) to improve the production of XOS and the enzymolysis efficiency in this study. We systematically studied the effects of pretreatment temperature, CO_2_ pressure and reaction time on the production of XOS, and analyzed the impact of CO_2_ on the enzymatic hydrolysis of solid residues. The composition of various pretreated substrates, supernatant, as well as subsequent enzymatic hydrolysates and residues was investigated. The synergistic mechanism of subcritical CO_2_ and inorganic ions in seawater was also studied. The co-production of functional oligosaccharides and fermentable sugars provides a promising method for the comprehensive utilization of SCB.

## Materials and methods

### Materials

SCB was obtained from Guitang Co., Ltd. (Guangxi, China). It was washed with tap water, then dried and ground to 40-mesh size, and finally stored in a sealed place at room temperature for further use. The chemical composition of SCB consisted of 37.61 ± 0.23% glucan, 21.87 ± 0.17% xylan, 20.60 ± 0.20% acid-insoluble lignin (AIL) and 2.30 ± 0.22% ash. The seawater was taken from Qingdao (Shandong, China), sterilized at 121 °C and passed through a 0.22-μm filter, and stored at 4 °C for subsequent use.

All the quantitative standard products such as glucose, xylose (X_1_), xylobiose (X_2_), xylotriose (X_3_), xylotetraose (X_4_), xylopentaose (X_5_), furfural (FF), 5- hydroxymethylfurfural (HMF) and acetic acid (AA) were purchased from Sigma, USA. Cellulase purchased from Novozymes (Copenhagen, Denmark), and its filter paper activity was 145.2 U/mL, endo-1, 4-β-d-glucanase activity and β-glucosidase activity were 3256.0 U/mL and 2774.2 IU/mL, respectively.

### *Subcritical CO*_*2*_*-assisted seawater pretreatment of sugarcane bagasse*

The SCSP of SCB was carried out in a 100 mL multifunctional stainless-steel reaction kettle (TGYF-C), which was automatically heated, stirred and timed. Based on the experiments we have studied, the pretreatment temperature was set as 135, 145, 155 and 165 °C, the reaction time was set as 5, 20, 35 and 50 min, and the CO_2_ pressure was 0, 1, 2 and 3 MPa (Zhang et al. 2020b). In addition, the yield of XOS as the objective, the L_9_ (3^3^) orthogonal experiment was designed with the pretreatment temperature, CO_2_ pressure and reaction time as factors (Table 1). The conditions for pretreatment were determined using the severity parameter (SP), defined according to Eq. (1):

**Table 1:** L_9_ (3^3^) factor and level of orthogonal experiment

Levels	*A* Pretreatment temperature (°C)	*B* CO_2_ pressure (MPa)	*C* Reaction time (min)
1	145	1	5
2	155	2	20
3	165	3	35

1 $$\mathrm{SP}=\mathrm{log}\left\{{\int }_{0}^{t}{\mathrm{p}}^{\mathrm{n}}{\mathrm{e}}^{\left(\frac{T-100}{14.75}\right)}\mathrm{dt}\right\},$$

where *t* is the reaction time (min), *T* is the pretreatment temperature (°C), 14.75 is an empirical parameter related to temperature and activation energy, P is the CO_2_ pressure (MPa), and n is an arbitrary constant (0.849 for acidic medium).

Raw SCB (5 g) and seawater (50 mL) were successively added into the reactor tank, and then the kettle cover was covered. CO_2_ was inserted into the gas inlet of the reactor, and the method was as follows. First, a small amount of gas was continuously injected for 2–3 min to eliminate the air in the reactor to ensure that the reactor was filled with CO_2_. Then, CO_2_ gas was continuously injected to achieve the required pressure and the gas outlet was closed. Next, the switch of the reaction kettle was turned on. When the reaction was over, the kettle body was cooled to room temperature quickly with tap water, the kettle cover opened and CO_2_ released rapidly. Finally, the resulting mixture was vacuumed and filtered to separate the solid and liquid components. The solid components were washed to neutral with excess deionized water and stored at 4 °C for subsequent cellulase hydrolysis and component analysis. The supernatant was stored at − 20 °C for further analysis of XOS (X_2_ − X_5_), monosaccharides (glucose and xylose) and by-products (FF and HMF). The XOS yield (*Y*) was calculated as follows from Eq. (2):

2 $$Y \left(\mathrm{\%}\right)=\frac{\mathrm{XOS\, in\, the\, supernatant }\left(\mathrm{g}\right)}{\mathrm{Xylan \, in \, raw \,SCB }\left(\mathrm{g}\right)}\times 100\%.$$

### Enzymatic hydrolysis of the solid residues

Enzymatic hydrolysis experiments were carried out on the untreated SCB and the pretreated solid residue in a 50-mL centrifuge tube, which was placed in a vibrating incubator at 48 °C and reacted at 150 rpm for 72 h. Substrate concentration was 5%, cellulase dosage was 30 FPU/g cellulose, and acetic acid–sodium acetate buffer (pH 4.8) was used as enzymatic hydrolysis system. Samples were taken at 4 and 72 h, and centrifuged to obtain supernatant for sugar analysis. The enzymatic hydrolysis residues were dried and stored for future analysis. The glucose yield (%) of pretreated solid residues after 72 h enzymatic hydrolysis is calculated as Eq. (3):

3 $$\mathrm{Glucose }\left(\mathrm{\%}\right)=\frac{\mathrm{Glucose \,after \,the \, enzymatic\, hydrolysis }\left(\mathrm{g}\right)\times 0.9}{\mathrm{Glucan\, in\, pretreated\, solid\, residues }\left(\mathrm{g}\right)}\times 100\%.$$

### High-performance liquid chromatography analysis

According to the method of the National Renewable Energy Laboratory (NREL) (Sluiter et al. 2008), the chemical composition (glucan, xylan, and AIL) of SCB and solid residues under different pretreatment conditions was determined. The sugars of raw SCB and liquid after enzymatic hydrolysis were determined by high-performance liquid chromatography (HPLC) (Waters e2695, USA) system with an Aminex HPX-87P column (300 × 7.8 mm; Bio-Rad Laboratories, USA) at 85 °C with a refractive index detector (RID) at 35 °C, and ultra-pure water at 0.6 mL/min was used as the mobile phase. In addition, the concentrations of by-products (AA, FF, HMF) after pretreatment were measured by HPLC with an HPX-87H column (300 × 7.8 mm; Bio-Rad Laboratories, USA) at 65 °C, using 5 mM H_2_SO_4_ as the eluent. Supernatant after SCSP was determined XOS content by a Sugar-Pak 1 column at 85 °C with ultra-pure water as the mobile phase at a flow rate of 0.5 mL/min. All liquids are filtered with the 0.22-μm filter before detection.

### Characterization methods of raw materials and solid residues

Physical adsorption analysis was performed using an automatic specific surface characteristics analyzer (Autosorb-IQ, Canta, USA) to evaluate the specific surface area and pore size distribution characteristics of samples. Test type was mesoporous, adsorption gas was nitrogen (N_2_), temperature was 120 °C, and degassing time was 3 h. The specific surface area and pore size distribution of all samples were calculated by Brunauer–Emmett–Teller (BET) equation, and the average pore size and pore volume were obtained (Iruretagoyena et al. 2020).

Surface morphology was analyzed by scanning electron microscopy (SEM) (SU8010, Japan). The samples were treated with gold spray in a vacuum and attached to the electron microscope sample table. The acceleration voltage of the electron beam was 5 kV.

The crystallinity of SCB and pretreated substrate were examined by X-ray diffraction (XRD) analysis (Rigaku Ultima IV diffractometer, Japan). The test parameters were as follows: the scanning range was in a 2 θ range between 10° and 80°, scanning speed was 5°/min, the scanning voltage and current was 40 kV and 40 mA. The crystallinity index (CrI) was calculated using Eq. (4):

4 $$\mathrm{CrI }\left(\mathrm{\%}\right)=\frac{{I}_{002}-{I}_{am}}{{I}_{002}}\times 100\mathrm{\%},$$

where *I*_*002*_ is the scattered intensity at a peak of around 22.5° and *I*_*am*_ is the scattered intensity of the amorphous portion around 18.1° (Segal et al. 1959).

## Results and discussion

### Reaction mechanisms of subcritical CO_2_-assisted seawater pretreatment on hemicellulose degradation

According to previous studies, the mechanism of CO_2_-assisted seawater pretreatment was speculated as follows: during the hydrothermal pretreatment at high temperature, the water was ionized into hydrogen ions, which can act as catalysts in acidic hydrolysis reactions, leading to degradation and dissolution of hemicelluloses (Wu et al. 2021a). In addition, complex ions in seawater first penetrated the SCB, and metal ions such as Mg^2+^ and then acted as Lewis acids combined with hydronium ion to break the glycosidic bonds of xylan, with Cl^−^ further converting xylo-oligomers into short-chain xylo-oligomers, simultaneously H_2_CO_3_ and acetic acid produced during the process assisted in this conversion (Zhang et al. 2020b). [M(H_2_O)n]^z+^ is the generic formula of metal ions ligand complexes, where M is the metal ion, z is the oxidation state of the cation, and n is the solvation number usually ranging from 4 to 9. The metal cations can play the role of Lewis acids and facilitate the breakage of glycosidic linkages (C–O–C) (Loow et al. 2015; Kamireddy et al. 2013; Zhang et al. 2019). The whole process involved chemical reactions are as follows Eqs. (5)−(8):

5 $${\text{H}}_{{2}} {\text{O + }} {\text{H}}_{{2}} {\text{O}} \rightleftarrows {\text{H}}_{{3}} {\text{O}}^{ + } + {\text{OH}}^{ - }$$

6 $${\text{H}}_{{2}} {\text{O}} + {\text{CO}}_{{2}} \rightleftarrows {\text{HCO}}_{{3}}^{ + } + {\text{OH}}^{ - } \rightleftarrows {\text{CO}}_{{3}}^{{{2} - }} + {\text{2H}}^{ + }$$

7 $$\left[ {{\text{M}}\left( {{\text{H}}_{{2}} {\text{O}}_{{6}} } \right)} \right]^{{{2} + }} + {\text{H}}_{{2}} {\text{O}} \rightleftarrows \left[ {{\text{M}}\left( {{\text{H}}_{{2}} {\text{O}}} \right)_{{5}} {\text{OH}}} \right]^{ + } + {\text{H}}_{{3}} {\text{O}}^{ + }$$

8 $$\left[ {{\text{M}}\left( {{\text{H}}_{{2}} {\text{O}}_{{6}} } \right)} \right]^{{{3} + }} + {\text{H}}_{{2}} {\text{O}} \rightleftarrows \left[ {{\text{M}}\left( {{\text{H}}_{{2}} {\text{O}}} \right)_{{5}} {\text{OH}}} \right]^{{{2} + }} + {\text{H}}_{{3}} {\text{O}}^{ + } ,$$

where M is the metal ion.

### Orthogonal experimental results of XOS yield

The optimum pretreatment temperature (155 °C), CO_2_ pressure (2 MPa) and reaction time (5 min) were determined by a single factor experiment (Additional file 1: Figs. S1–S3). Under this pretreatment condition, the yield of XOS reached 58.13%. In order to optimize the pretreatment conditions and obtain a higher XOS yield, the orthogonal experiment was carried out with XOS yield as the index.

As can be seen from the data in Table 2, the primary and secondary factors affecting the yield of XOS were *A* > *C* > *B*. That was, pretreatment temperature had the most significant influence on XOS yield, followed by reaction time and CO_2_ pressure. Meanwhile, the optimal pretreatment process was *A*_3_*B*_2_*C*_1_ (165 °C-2 MPa-5 min) according to *K* value, under which the yield of XOS was 68.23%.

**Table 2:** The arrangement and results of orthogonal experiment

Number	*A* (°C)	*B* (MPa)	*C* (min)	*Y*^a^ (%)	SP
1	145	1	5	17.16	2.02
2	145	2	20	19.14	2.88
3	145	3	35	20.81	3.27
4	155	1	20	59.32	2.92
5	155	2	35	53.95	3.42
6	155	3	5	50.26	2.72
7	165	1	35	60.04	3.46
8	165	2	5	68.23	2.87
9	165	3	20	51.67	3.62
*K*_*1*_	19.04	45.51	45.22		
*K*_*2*_	54.51	47.11	43.38		
*K*_*3*_	59.98	40.91	44.93		
*R*	45.45	2.65	3.23		

SP: severity parameter

^a^*Y* is the XOS (X_2_ − X_5_) yield

Table 2 shows the SP of reaction conditions for SCSP. Different from our previous exploration of seawater hydrothermal pretreatment (Zhang et al. 2020b), the addition of CO_2_ in this study was a crucial factor for XOS production. It can be found that CO_2_ pressure effectively reduces the SP under the condition of obtaining a similar XOS yield. For example, in the SCSP process, when the yields of XOS were 59.32% (155 °C-1 MPa-20 min) and 68.23% (165 °C-2 MPa-5 min), respectively. The corresponding SP values were 2.92 and 2.87, respectively. However, in the seawater hydrothermal pretreatment process, the XOS yields were 60.58% (165 °C-70 min) and 67.12% (175 °C-30 min), the corresponding SP values were 3.76 and 3.68, respectively (Additional file 1: Table S1). In other words, under the same pretreatment temperature and reaction time, CO_2_ will promote the degradation of more hemicellulose to XOS, which significantly improves the yield of XOS.

### Effects of different pretreatment conditions on XOS and by-products

**Fig. 1:**
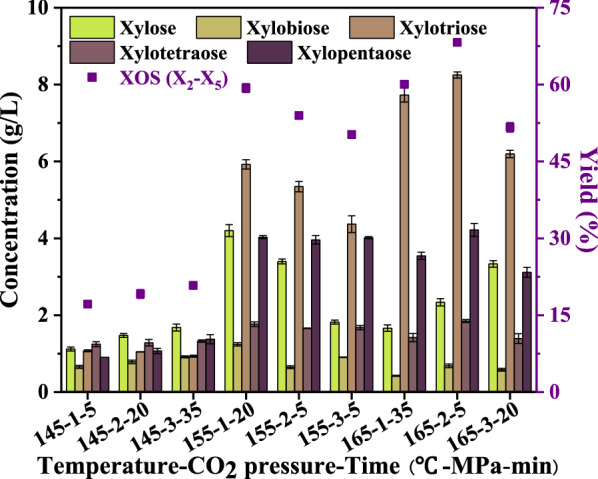
Xylose and XOS concentrations and XOS yield under different treatment conditions

Subcritical CO_2_-assisted seawater pretreatment, breaks down the linkages among hemicellulose, cellulose, and lignin of the SCB and releases or degrades the hemicellulose firstly (Liu et al. 2019). Pretreatment temperature, CO_2_ pressure and reaction time have different effects on the degradation of hemicellulose into XOS with different degrees of polymerization. The degree of polymerization of XOS varies under different pretreatment conditions. Still, the concentration of X_3_ was higher (Fig. 1), which inferred that the X_3_ was more stable and easier to form in these conditions, consistent with the study of Liu et al. (2019). When the pretreatment temperature was 145 °C, the concentration of XOS and xylose in the supernatant was generally low. With the increase of CO_2_ pressure and reaction time, the concentration of XOS and xylose increased, but the increasing trend was steady. However, when the pretreatment temperature was increased to 155 °C and 165 °C, the concentration of XOS increased significantly. When XOS (X_2_ − X_5_) yield was 59.32% (155 °C-1 MPa-20 min), this was consistent with the XOS yield under the same pretreatment conditions in the single factor experiment (Additional file 1: Fig. S2). Under the optimal condition (165 °C-2 MPa-5 min), the yield of XOS (X_2_ − X_5_) was 68.23%, the concentrations of X_2_, X_3_, X_4_ and X_5_ were 0.68 g/L, 8.25 g/L, 1.85 g/L and 4.22 g/L, respectively.

In addition, the influences of different solvents (seawater and freshwater) on the production of XOS in the pretreatment system were compared (Additional file 1: Fig. S5). It could be found that the yield of XOS in the seawater system was significantly higher than that in the freshwater system. The difference in XOS (X_2_ − X_5_) concentration was slight (0.48 g/L) at a lower temperature (145 °C) but significant (5.84 and 8.28 g/L) at higher temperatures (155 and 165 °C). In summary, it was proved once again that temperature was the most critical factor affecting the yield of XOS. In other words, the pretreatment must reach a specific temperature to obtain XOS with higher concentration and yield. The XOS yield in the seawater system increased by 37.88% compared with the freshwater system (165 °C-2 MPa-5 min). The results showed that the synergistic effect of seawater and CO_2_ was more substantial than that of freshwater and CO_2_. This phenomenon means that the ions in seawater and CO_2_ have an excellent synergistic effect.

**Fig. 2:**
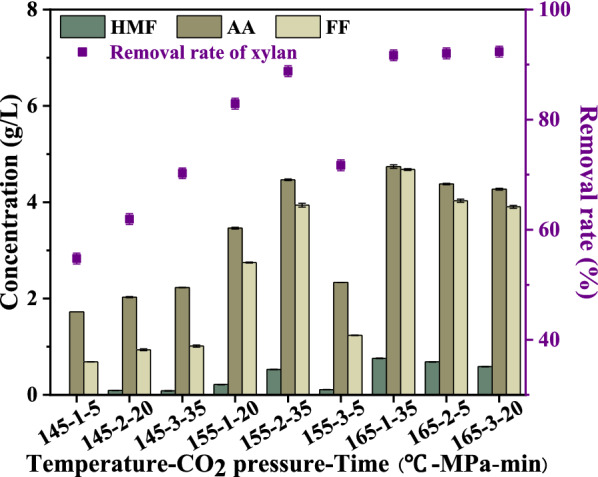
The concentration of by-products and removal rate of xylan under different treatment conditions

SP values and by-products (HMF, AA, and FF) concentrations indirectly reflected the intensity of pretreatment conditions (Table 2 and Fig. 2). The concentration of by-products and the removal rate of xylan were closely related to SP values. At the same temperature, the removal rate of xylan increased with the increase of SP value. For example, when the SP value increased from 2.02 to 3.27, the removal rate of xylan rose from 50.76 to 70.26%, and the yield of XOS increased from 17.16 to 20.81% at 145 °C. HMF and FF are degradation products of glucose and xylose. Under all pretreatment conditions, the concentration of HMF was lower than that of FF, indicating that xylose was more easily degraded (Fig. 1 and Fig. 2). In addition, the removal rate of xylan increased gradually with the deepening of pretreatment conditions, and the maximum xylan removal rate reached 92.35% (165 °C-3 MPa-20 min).

To some extent, the composition of solid residues after SCSP also explained the degradation sequence of lignin, cellulose and hemicellulose (Table 3). With the increase of pretreatment temperature, the xylan content in the solid residue decreased gradually. For example, an increase in pretreatment temperature from 135 °C to 165 °C resulted in a reduction in xylan content from 19.75% to 3.38%. But for the other two conditions (CO_2_ pressure and reaction time), the reduction in xylan content was to a lesser extent (Additional file 1: Table S2). When CO_2_ pressure increased from 1 to 3 MPa, xylan content decreased only 3.71%; the reaction time was extended from 5 to 50 min, and the xylan content decreased by 3.63%. This result indicated that compared with the pretreatment temperature, the degradation effect of CO_2_ pressure and reaction time on xylan was weaker, consistent with the above conclusion (Table 2).

**Table 3:** The three components yield of sugarcane bagasse after SCSP of orthogonal experiment

*A* (°C)	*B* (MPa)	*C* (min)	Glucan^a^ (%)	Xylan^b^ (%)	AIL^c^ (%)
145	1	5	51.10 ± 0.29	13.56 ± 0.06	19.70 ± 3.97
	2	20	52.65 ± 0.10	11.68 ± 0.02	25.00 ± 0.67
	3	35	53.32 ± 0.59	9.60 ± 0.54	25.80 ± 0.80
155	1	20	58.17 ± 0.75	5.85 ± 0.02	26.64 ± 1.36
	2	35	56.96 ± 0.58	3.91 ± 0.03	31.80 ± 2.73
	3	5	53.07 ± 2.76	9.55 ± 0.40	24.97 ± 1.23
165	1	35	57.32 ± 0.68	2.95 ± 0.03	28.64 ± 4.16
	2	5	56.29 ± 0.20	2.85 ± 0.03	32.40 ± 0.87
	3	20	56.91 ± 0.45	2.75 ± 0.02	30.97 ± 2.64

All values are mean ± standard deviation of two replicate determinations.

^a^Glucan yield = (glucan in solid residues (%) × solid residue (g)/glucan in raw substrate (g)) × 100%

^b^Xylan yield = (xylan in solid residues (%) × solid residue (g)/xylan in raw substrate (g)) × 100%

^c^AIL yield = (AIL in solid residues (%) × solid residue (g)/AIL in raw substrate (g)) × 100%

In conclusion, the results of single factor and orthogonal experiments showed that the X_3_ content accounted for the largest proportion of XOS under most SCSP processes. However, the X_4_ content accounted for the largest proportion of XOS in the seawater hydrothermal pretreatment process. The results showed an internal relationship between CO_2_ and mixed ions in seawater, which can better promote the transformation of XOS from a high degree of polymerization to a low degree of polymerization. Previous study has shown that XOS with average DP of 3–4 has better prebiotic effect than XOS with DP of 5–6 (Moura et al. 2007), which further proves the advantage of SCSP.

### Characterization of raw materials and pretreated solid residues

**Fig. 3:**
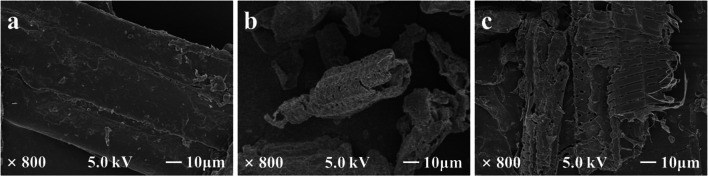
SEM of SCB before and after pretreatment. **a** Raw SCB, **b** 175 °C-30 min, **c** 155 °C-1 MPa-20 min

The morphological structure of raw SCB and pretreated solid residues (seawater hydrothermal pretreatment and SCSP) were displayed by SEM (Fig. 3). Raw SCB showed a smooth, complete, solid, continuous, flat surface and highly fibrillary state. However, due to the degradation of hemicellulose and lignin and the decomposition between lignin and carbohydrates, the pretreated SCB structure showed separation and disorder, and the surface became rough. After pretreatment, many large and small holes appeared on the SCB surface, especially in the solid residue by SCSP. This phenomenon can be explained as follows: CO_2_ increased the pressure of the reaction system; CO_2_ gas and water reacted to generate H_2_CO_3_ and penetrated into the internal structure of SCB. After the reaction finished, rapid decompression made CO_2_ gas released from SCB and expanded the pores on the fiber surface, and made the pretreated SCB have a higher pore area and porosity (Ge et al. 2020). SCSP removed a large amount of hemicellulose and part of lignin, destroyed the internal structure or carbohydrate composite structure of SCB, loosened the surface structure, and leaked more cellulose for the accessibility of enzymes (Ge et al. 2020; Huang et al. 2018; Lin et al. 2020).

The BET surface area, micropore surface area, total pore volume, and average pore size of the SCB and solids pretreated by different pretreatment processes are shown in Table 4. The BET surface area of SCB after seawater hydrothermal pretreatment (175 °C-30 min) increased from 2.47 to 5.72 m^2^/g, and the average pore size increased from 4.74 to 19.30 nm. After SCSP (155 °C-1 MPa-20 min), the BET surface area increased by 218.57% (from 2.47 m^2^/g to 7.88 m^2^/g), the total pore volume increased by 200% (from 0.01 cm^3^/g to 0.03 cm^3^/g), and the average pore diameter increased by 585.44% (from 4.74 nm to 32.49 nm). The experimental results were consistent with the above SEM phenomena. It was proved that CO_2_ gas with a certain pressure increased the porosity of SCB and was more conducive to the penetration of inorganic salts, thus promoting the reaction of XOS production. At the same time, the contact area between cellulase and substrate was increased in subsequent enzymatic hydrolysis.

**Table 4:** BET surface area, total pore volume and average pore size of SCB and solids pretreated by different processes

Samples	BET surface area (m^2^/g)	Total pore volume (cm^3^/g)	Average pore size (nm)
Raw SCB	2.47	0.01	4.74
175 °C-30 min	5.72	0.02	19.30
155 °C-1 MPa-20 min	7.88	0.03	32.49

**Fig. 4:**
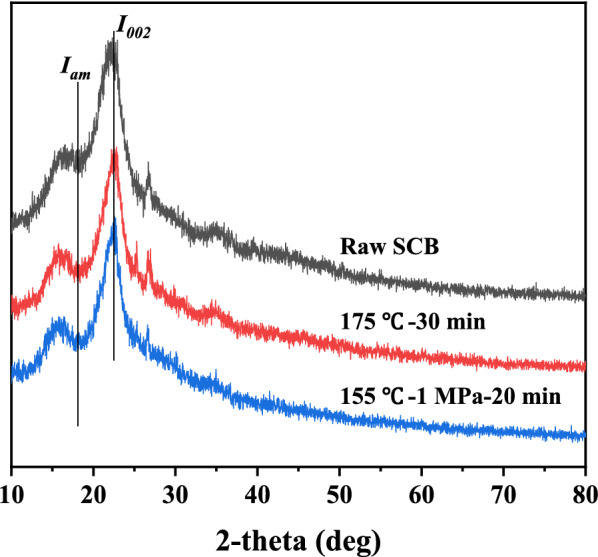
X-ray diffraction (XRD) of SCB and solids pretreated by different processes

XRD analysis was performed for the untreated and pretreated SCB to study the crystallinity index (CrI) from the diffraction patterns. The changes of the crystallization region after pretreatment can be seen in Fig. 4. In the pretreatment process, two factors determine the crystallinity of pretreated SCB: decrystallizing by swelling and dissolving the crystalline fraction of cellulose, and increasing CrI by reducing amorphous cellulose, lignin and hemicellulose (Chourasia et al. 2021). CrI calculated for untreated SCB was found to be 36%, while seawater hydrothermal pretreated SCB was 52%, and SCSP pretreated SCB was 61%. The addition of CO_2_ in the reaction process made more hemicellulose removed. Therefore, the results showed an increase in CrI after pretreatment implying removal of amorphous fractions of SCB. These results thus showed the consistency with SCB composition analysis, SEM and BET results before and after treatment.

### Effects of different pretreatment conditions on enzymatic hydrolysis

It has been proved that SCSP can reduce the reaction conditions and produce high-yield XOS. At the same time, in order to make better use of the solid residue after pretreatment, the cellulase hydrolysis experiment of the solid residue under partial pretreatment conditions was carried out. The presence of CO_2_ increased the pores in the solid residue, to increase the specific surface area of contact between cellulase and cellulose. Moreover, the pretreated solid residue was rich in cellulose, and more glucose products can be obtained during enzymatic hydrolysis and thus, the utilization rate of solid residue was increased.

**Fig. 5:**
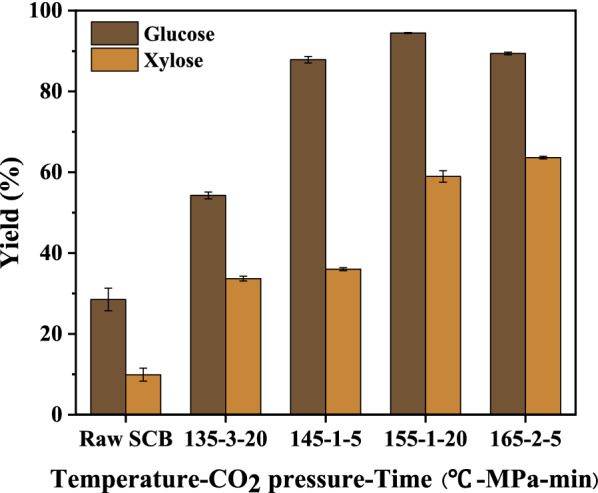
The yield of fermentable sugar produced by enzymatic hydrolysis

The results of enzymatic hydrolysis of solid residues are shown in Fig. 5. SCSP process improved the yield of enzymatic hydrolysis of glucose. For example, the glucose yield of raw SCB was 28.52% without any treatment, while the highest glucose yield of pretreated residue is 94.45% (155 °C-1 MPa-20 min). In addition, it had been proved from the above discussion that the temperature was the main factor affecting the yield of XOS, so the temperature was selected as a variable to pretreat the residue for enzymatic hydrolysis. It can be seen that the glucose yield was different at different temperatures, but there was an obvious boundary (145 °C). Before this boundary, the temperature had a significant effect on the glucose yield, and the influence decreased after this boundary. For example, when the pretreatment temperature was 135 °C, although the CO_2_ pressure was high (3 MPa) and the reaction time was long (20 min), the yield of glucose was still low (54.26%); when the pretreatment temperature increased to 145 °C, the CO_2_ pressure was 1 MPa and the reaction time was 5 min, the yield of glucose increased significantly (87.86%); the increase of glucose yield was not evident when the temperature continued to grow. The appropriate pretreatment temperature was the key factor to improve the yield of glucose. In other words, only after the pretreatment reaches a specific temperature can a large amount of hemicellulose and some lignin be removed and more cellulose is exposed, to promote the enzymatic hydrolysis reaction (Liu et al. 2019). Similarly, the changing trend of xylose yield was similar to that of glucose. However, the xylose yield of enzymatic hydrolysis of solid residue was generally low. Most of the hemicellulose was degraded after pretreatment, and few can be retained in the solid residue for enzymatic hydrolysis. In summary, SCSP can substantially increase the cellulolytic digestibility of SCB and increase the yield of glucose (Zhang et al. 2017).

### Competitive advantage analysis of subcritical CO_2_-assisted seawater pretreatment

Table 5 shows the comparison of XOS and fermentable sugars produced by some pretreatment methods. It can be seen that the yield of XOS and fermentable sugar produced is different due to different raw materials and pretreatment methods. SCSP can produce XOS with high yield at lower temperature and shorter reaction time compared with other methods. Similarly, the type of enzyme and enzymatic hydrolysis conditions also affect the yield of fermentable sugars (relative to the content of glucan/xylan in the raw material). Compared with the other results, through the ingenious coupling of seawater and CO_2_, this study achieved a higher XOS yield under lower pretreatment conditions (165 °C-2 MPa-5 min), promoted the subsequent enzymatic hydrolysis reaction and obtained 94.45% glucose.

**Table 5:** XOS yield and enzymatic hydrolysis performance under different pretreatment strategies

Raw material	Pretreatment conditions	XOS yield	Enzyme	Enzymatic hydrolysis conditions	Yield	Ref
Poplar sawdust	6.5% acetic acid, 170 °C, 27 min	(DP 2–6) 36.0%	C2730, Celluclast® 1.5 L	50 °C, pH 4.8, 20 FPU/g cellulose, 108 h	Glucose: 51.0%	Huang et al. (2018)
Poplar	1% H_2_SO_4_ delignification; 2% lactic acid, 170 °C, 30 min	(DP 2–6) 42.7%	Cellulase (Cellic CTec2)	50 °C, pH 5.0, 10 mg cellulase protein/g dry matter, 72 h	Glucose: 88.9%; xylose: 66.2%	Yang et al. (2019)
SCB: white birch = (65:35)	Autohydrolysis; 200 °C, 10 min	(DP 2–5) 52.99%	Cellulase (Cellic CTec2)	50 °C, pH 4.8, 18 FPU/g cellulose, 72 h	Glucose: 84.74%; xylose: 72.54%	Zhang et al. (2018)
SCB	0.1 M MgCl_2_, 180 °C, 10 min	(DP 2–5) 53.79%	Cellulase (Cellic CTec2)	50 °C, pH 4.8, 18 FPU/g cellulose, 72 h	Glucose: 71.62%; xylose: 66.75%	Zhang et al. (2019)
Corn straw	5 MPa CO_2_, 170 °C, 40 min	(DP 2–5) 40%	Commercial cellulase	50 °C, pH 4.8, 20 FPU/g cellulose, 72 h	Glucose: 90.2%; xylose: 81.7%	(Liu et al. 2019)
SCB	Seawater hydrothermal pretreatment; 165 °C, 2 MPa CO_2_, 5 min	(DP 2 − 5) 68.23%	Commercial cellulase	48 °C, pH 4.8, 30 FPU/g cellulose, 72 h	Glucose: 94.45%	This study

### Material balance at optimal pretreatment conditions

**Fig. 6:**
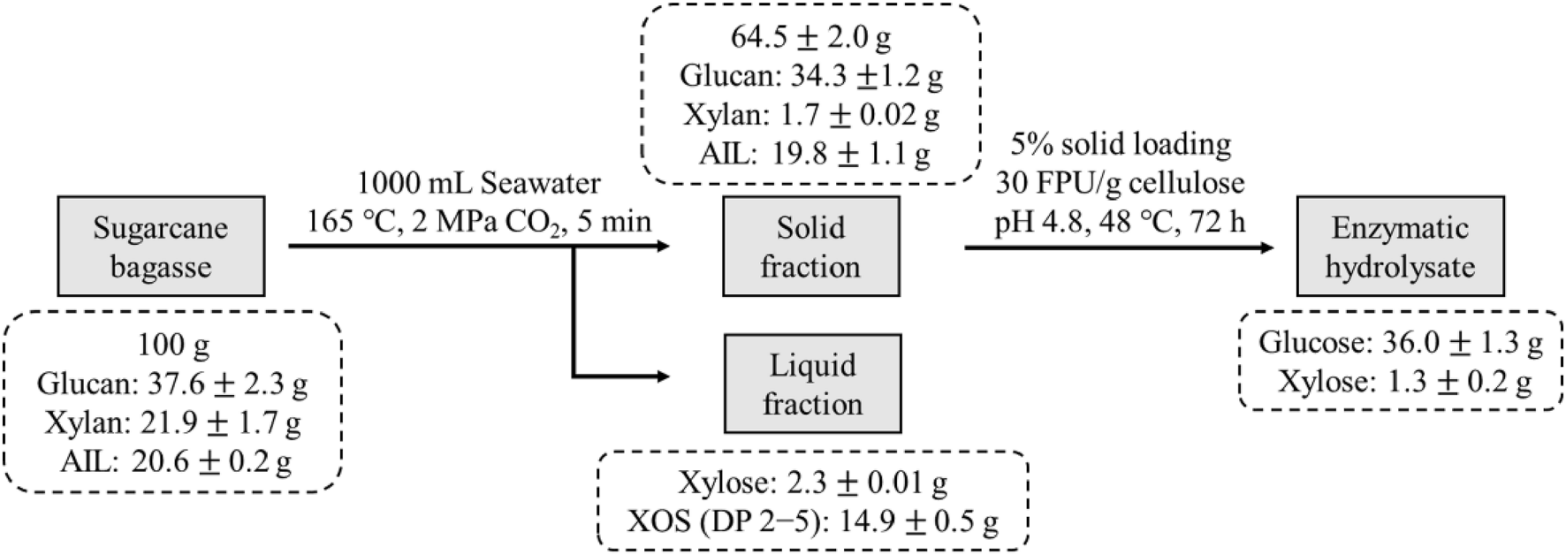
Material balances for subcritical CO_2_-assisted seawater pretreatment and subsequent enzymatic hydrolysis

In this study, XOS and fermentable sugars were co-produced efficiently by SCSP and enzymatic hydrolysis. Figure 6 summarizes the material balance based on 100 g dry SCB under the optimum conditions. From 100 g of dry raw SCB (comprising 37.6 g glucan, 21.9 g xylan, and 20.6 g AIL), 64.5 g of SCB containing 34.3 g glucan, 1.7 g xylan and 19.8 g AIL were recovered by SCSP, which indicated most of hemicellulose were degraded. At the same time, 2.3 g xylose and 14.9 g XOS (DP 2 − 5) were released from the pretreatment liquid. After enzymatic hydrolysis, about 36.0 g glucose and 1.3 g xylose were recovered. The yield of glucose was 94.45%, accounting for 86.17% of the glucose content in the raw SCB. The results showed that a small amount of sugar was lost during pretreatment and washing, most of the sugar was retained.

## Conclusions

In this study, SCSP was explored for transforming SCB into XOS and fermentable sugar. Seawater, the only reaction solvent, saves freshwater resources. A small amount of subcritical CO_2_ reduces the pretreatment temperature and reaction time, thus reducing the cost. The process is green and pollution-free without adding any chemical reagent, and the highest yield of XOS is 68.23% (165 °C-2 MPa-5 min), and the highest yield of glucose after enzymatic hydrolysis is 94.45%. The combined production of XOS and glucose realizes the high-value utilization of SCB. Future work can further study the full utilization of lignocellulosic biomass and expand the application range of each component.

### Supplementary Information

**Additional file 1: Fig. S1.** Effect of pretreatment temperature on the yield **(a)** and concentration **(b)** of xylose and XOS. **Fig. S2.** Effect of CO_2_ pressure on the yield **(a)** and concentration **(b)** of xylose and XOS. **Fig. S3.** Effect of reaction time on the yield **(a)** and concentration **(b)** of xylose and XOS. **Fig. S4.** Effect of different conditions on the concentration of by-products. **Fig. S5.** Effect of different solvents (seawater and freshwater) on the yield and concentration of XOS. **Table S1.** The severity parameter (SP) about seawater hydrothermal pretreatment. **Table S2.** The three components yield of sugarcane bagasse after subcritical CO_2_-assisted seawater pretreatment of single factor experiments.

## Data Availability

Not applicable.

## Abbreviations

AA: Acetic acid
AIL: Acid-insoluble lignin
FF: Furfural
HMF: 5-Hydroxymethylfurfural
LCC: Lignin-carbohydrate complex
SCB: Sugarcane bagasse
SCSP: Subcritical CO_2_-assisted seawater pretreatment
X_1_: Xylose
X_2_: Xylobiose
X_3_: Xylotriose
X_4_: Xylotetraose
X_5_: Xylopentaose
XOS: Xylo-oligosaccharide
