# Supplementary material for: Coproduction of xylo-oligosaccharides and glucose from sugarcane bagasse in subcritical CO2-assisted seawater system
Source: Bioresour Bioprocess. 2022 Mar 28;9(1):34. doi: 10.1186/s40643-022-00525-3 (PMC10991134; doi:10.1186/s40643-022-00525-3)
Supplement: Supplementary file 1 — Additional file 1: Fig. S1. Effect of pretreatment temperature on the yield (a) and concentration (b) of xylose and XOS. Fig. S2. Effect of CO2 pressure on the yield (a) and concentration (b) of xylose and XOS. Fig. S3. Effect of reaction time on the yield (a) and concentration (b) of xylose and XOS. Fig. S4. Effect of different conditions on the concentration of by-products. Fig. S5. Effect of different solvents (seawater and freshwater) on the yield and concentration of XOS. Table S1. The severity parameter (SP) about seawater hydrothermal pretreatment. Table S2. The three components yield of sugarcane bagasse after subcritical CO2-assisted seawater pretreatment of single factor experiments. [file 40643_2022_525_MOESM1_ESM.docx]

**Coproduction** **xylo-oligosaccharides and glucose** **from sugarcane bagasse in subcritical CO_2_-assisted seawater system**

Leping Zhang^1^, Xiankun Zhang^1^, Fuhou Lei^2^, Jianxin Jiang^1^*, Li Ji^1^*

^1^ Department of Chemistry and Chemical Engineering, MOE Engineering Research Center of Forestry Biomass Materials and Bioenergy, Beijing Forestry University, Beijing, 100083, China

^2^ Key Laboratory of Chemistry and Engineering of Forest Products, State Ethnic Affairs Commission, Guangxi Key Laboratory of Chemistry and Engineering of Forest Products, School of Chemistry and Chemical Engineering, Guangxi University for Nationalities, Nanning 530006, China

*Correspondence: jiangjx2004@hotmail.com (J. J.) bjfu090524116@163.com (L. J.)

**Supporting information**

**Fig. S1.** Effect of pretreatment temperature on the yield **(a)** and concentration **(b)** of xylose and XOS

**Fig. S2.** Effect of CO_2_ pressure on the yield **(a)** and concentration **(b)** of xylose and XOS

**Fig. S3.** Effect of reaction time on the yield **(a)** and concentration **(b)** of xylose and XOS

**Figure S4.** Effect of different conditions on the concentration of by-products

**Figure S5.** Effect of different solvents (seawater and freshwater) on the yield and concentration of XOS

**Table S1 The severity parameter (SP) about seawater hydrothermal pretreatment**

| Pretreatment temperature  (°C) | 165 | | | | 175 | | | | 185 | | | |
| --- | --- | --- | --- | --- | --- | --- | --- | --- | --- | --- | --- | --- |
| Reaction time  (min) | 10 | 30 | 50 | 70 | 10 | 30 | 50 | 70 | 10 | 30 | 50 | 70 |
| SP^a^ | 2.91 | 3.39 | 3.61 | 3.76 | 3.21 | 3.68 | 3.91 | 4.05 | 3.50 | 3.98 | 4.20 | 4.35 |

^a^ It's computed by the equation $SP=log\{\int_{0}^{t} e^{(\frac{T-100}{14.75})}\mathrm{dt}\}$.Where, t is the reaction time (min), T is the pretreatment temperature (°C), 14.75 is an empirical parameter related to temperature and activation energy.

**Table S2** **The three components yield of sugarcane bagasse after subcritical CO_2_-assisted seawater pretreatment of single factor experiments**

| **Conditions** | **Components analysis after pretreatment** | | |  |
| --- | --- | --- | --- | --- |
| **Pretreatment temperature-CO_2_ pressure-Reaction time (°C- MPa- min)** | **Glucan^a^ (%)** | **Xylan^b^ (%)** | **Acid insoluble lignin^c^ (%)** | **Xylan removal (%)** |
| 135-3-20 | 43.30 ± 1.65 | 19.75 ± 0.82 | 22.90 ± 0.17 | 22.17 ± 1.03 |
| 145-3-20 | 50.04 ± 0.83 | 14.30 ± 0.16 | 24.23 ± 1.70 | 52.26 ± 0.99 |
| 155-3-20 | 56.77 ± 1.32 | 3.58 ± 0.14 | 33.07 ± 0.26 | 89.55 ± 0.84 |
| 165-3-20 | 57.51 ± 0.43 | 3.38 ± 0.06 | 36.44 ± 0.43 | 92.35 ± 0.93 |
| 155-0-20 | 42.95 ± 0.64 | 21.79 ± 0.33 | 21.10 ± 1.30 | 15.90 ± 0.99 |
| 155-1-20 | 56.23 ± 0.38 | 7.29 ± 0.03 | 28.87 ± 0.66 | 77.41 ± 1.00 |
| 155-2-20 | 56.12 ± 0.03 | 6.17 ± 0.00 | 29.80 ± 0.90 | 81.27 ± 1.00 |
| 155-3-20 | 56.77 ± 1.32 | 3.58 ± 0.14 | 33.07 ± 0.26 | 89.55 ± 0.84 |
| 155-2-5 | 55.43 ± 0.19 | 7.34 ± 0.02 | 29.20 ± 1.53 | 78.01 ± 1.00 |
| 155-2-20 | 56.12 ± 0.03 | 6.17 ± 0.00 | 29.80 ± 0.90 | 81.27 ± 1.00 |
| 155-2-35 | 56.23 ± 0.38 | 5.86 ± 0.09 | 25.90 ± 0.57 | 83.52 ± 0.99 |
| 155-2-50 | 56.61 ± 0.26 | 3.71 ± 0.02 | 32.20 ± 2.27 | 89.51 ± 1.00 |

All values are mean ± standard deviation of two replicate determinations.

^a^ Glucan yield = (Glucan in solid residues (%) × solid residue (g)/Glucan in raw substrate (g)) × 100%

^b^ Xylan yield = (Xylan in solid residues (%) × solid residue (g)/ Xylan in raw substrate (g)) × 100%

^c^ Acid insoluble lignin yield = (Acid insoluble lignin in solid residues (%) × solid residue (g)/ Acid insoluble lignin in raw substrate (g)) × 100%
